# Supplementary material for: Predation and fragmentation portrayed in the statistical structure of prey time series
Source: BMC Ecol. 2009 May 6;9:10. doi: 10.1186/1472-6785-9-10 (PMC2689204; doi:10.1186/1472-6785-9-10)
Supplement: Additional file 2 — Voles and related classes ODDox Documentation. ODDox documentation of the agent-based model (ALMaSS) applied by Hendrichsen et al. The documentation is started by activating main.html. [file 1472-6785-9-10-S2.zip › Vole_ODDox/_vole__all_8cpp.html]

ALMaSS ODDox: Vole\_all.cpp File Reference

- Main Page
- Related Pages
- Classes
- Files

# Vole\_all.cpp File Reference

---

## Detailed Description

**Vole\_all.cpp This file contains the code for all vole lifestage classes**   

by Chris J. Topping   
Version of 28th Jan 2001   
All rights reserved.   
  
With additions as noted in:   
April 2006   
November 2007   
Doxygen formatted comments in May 2008   

`#include "ls.h"`  
`#include "PopulationManager.h"`  
`#include "GeneticMaterial.h"`  
`#include "Vole_all.h"`  
`#include "VolePopulationManager.h"`  
`#include "MovementMap.h"`  
`#include "BatchALMaSS/BoostRandomGenerators.h"`  

|  |  |
| --- | --- |
|  |  |
| Defines | |
| #define | \_\_SpecificPesticideEffectsVinclozolinLike\_\_ |
| #define | \_CRT\_SECURE\_NO\_DEPRECATE |
| Functions | |
| CfgFloat | cfg\_extradispmort ("VOLEDISPMORT", CFG\_CUSTOM, 0.0025) |
| static CfgFloat | cfg\_PesticideAccumulationThreshold ("VOLE\_PESTICDEACCUMULATIONTHRESHOLD", CFG\_CUSTOM, 25.0) |
| static CfgInt | cfg\_vole\_max\_age ("VOLE\_MAX\_AGE", CFG\_CUSTOM, 547) |
| Variables | |
| CfgBool | cfg\_CIPEGridOutput\_used |
| CfgInt | cfg\_productapplicendyear |
| CfgInt | cfg\_productapplicstartyear |
| CfgBool | cfg\_ReallyBigOutputMonthly\_used |
| CfgBool | cfg\_RipleysOutput\_used |
| const double | DailyMortChance = 0.004 |
| const double | DaysAtMaxGrowth = 90.0 |
| const unsigned | FemaleMovement = 50 |
| const double | FemaleResourceReq [13] |
| const unsigned | FemaleTerritoryRange = 8 |
| const unsigned | FemaleTerritoryRangeSlope = (MaxFemaleTerrSize-FemaleTerritoryRange)/(MaxWeightF-MinReproWeightF) |
| const int | FemNoMature = September |
| const double | FHabQualThreshold1 = 1.00\*MinFVoleHabQual |
| const double | FHabQualThreshold2 = 1.50\*MinFVoleHabQual |
| const double | FHabQualThreshold3 = 2.00\*MinFVoleHabQual |
| double | g\_extradispmort |
| boost::variate\_generator  < base\_generator\_type   &, boost::uniform\_real<> > | g\_rand\_uni |
| double | g\_SpeedyDivides [2001] |
| const int | GrowStopDate = August |
| const double | growthperdayF = (MaxWeightF-WeanedWeight)/DaysAtMaxGrowth |
| const double | growthperdayM = (MaxWeightM-WeanedWeight)/DaysAtMaxGrowth |
| const double | InfanticideChanceByAge [9] = {0.97,0.86,0.75,0.64,0.54,0.43,0.32,0.21,0.11} |
| CfgBool | l\_pest\_enable\_pesticide\_engine |
| const unsigned | MaleMovement [4] = {10,40,70,110} |
| const int | MaleReproductFinish = October |
| const unsigned | MaleTerritoryRange = 12 |
| const unsigned | MaleTerritoryRangeSlope |
| const double | MaturationChance [5] = {0.020,0.100,0.200,0.500,1.000} |
| const unsigned | MaxFemaleTerrSize = 16 |
| const unsigned | MaxMaleTerrSize = 20 |
| const int | MaxStarvationDays = 21 |
| const int | MaxWeightF = 55 |
| const int | MaxWeightM = 60 |
| const double | MHabQualThreshold1 = 1.00\*MinMVoleHabQual |
| const double | MHabQualThreshold2 = 1.50\*MinMVoleHabQual |
| const double | MHabQualThreshold3 = 2.00\*MinMVoleHabQual |
| const unsigned | MinFemaleMovement = 5 |
| const double | MinFVoleHabQual = 4.0\*FemaleTerritoryRange\*FemaleTerritoryRange\*1.0 |
| const unsigned | MinMaleMovement = 5 |
| const double | MinMVoleHabQual = 4.0\*MaleTerritoryRange\*MaleTerritoryRange\*1.0 |
| const int | MinReproAgeF = 19 |
| const int | MinReproAgeM = 40 |
| const int | MinReproWeightF = 20 |
| const int | MinReproWeightM = 40 |
| double | MoveToLessFavourable |
| const unsigned | TheGestationPeriod = 21 |
| const int | Vector\_x [8] = { 0, 1,1,1,0,-1,-1,-1} |
| const int | Vector\_y [8] = { -1,-1,0,1,1, 1, 0,-1} |
| const double | VoleHarvestMort = 0.25 |
| const double | VoleHerbicicideMort = 0.0 |
| const double | VoleInsecticideMort = 0.0 |
| const unsigned | VoleMoveInterval = 30 |
| const double | VolePigGrazingMort = 0.25 |
| const double | VoleSoilCultivationMort = 0.75 |
| const double | VoleStriglingMort = 0.50 |
| const unsigned | WeanedAge = 13 |
| const int | WeanedWeight = 5 |

---

## Define Documentation

#define \_\_SpecificPesticideEffectsVinclozolinLike\_\_

#define \_CRT\_SECURE\_NO\_DEPRECATE

---

## Function Documentation

|  |  |  |  |  |
| --- | --- | --- | --- | --- |
| CfgFloat cfg\_extradispmort | ( | "VOLEDISPMORT" | , |  |
|  |  | CFG\_CUSTOM | , |  |
|  |  | 0. | *0025* |  |
|  | ) |  |  |  |

|  |  |  |  |  |
| --- | --- | --- | --- | --- |
| static CfgFloat cfg\_PesticideAccumulationThreshold | ( | "VOLE\_PESTICDEACCUMULATIONTHRESHOLD" | , |  |
|  |  | CFG\_CUSTOM | , |  |
|  |  | 25. | *0* |  |
|  | ) |  |  | `[static]` |

Referenced by Vole\_Female::st\_UpdateGestation().

|  |  |  |  |  |
| --- | --- | --- | --- | --- |
| static CfgInt cfg\_vole\_max\_age | ( | "VOLE\_MAX\_AGE" | , |  |
|  |  | CFG\_CUSTOM | , |  |
|  |  | 547 |  |  |
|  | ) |  |  | `[static]` |

---

## Variable Documentation

CfgBool cfg\_CIPEGridOutput\_used

CfgInt cfg\_productapplicendyear

Toxicological variable for specific pesticide effects

Referenced by Vole\_Female::st\_UpdateGestation().

CfgInt cfg\_productapplicstartyear

Toxicological variable for specific pesticide effects

Referenced by Vole\_Female::st\_UpdateGestation().

CfgBool cfg\_ReallyBigOutputMonthly\_used

CfgBool cfg\_RipleysOutput\_used

const double DailyMortChance = 0.004

Mortality per day as a background mortality encompassing all things not directly modelled. This may include specialist predators if they are not simulated - if they are then reduce this value

const double DaysAtMaxGrowth = 90.0

How long it takes to get to max weight at max rate of growth

const unsigned FemaleMovement = 50

Max distance between fix distance in Mols radiotracking data was 48m

const double FemaleResourceReq[13]

**Initial value:**

```
                  {
                  0,
                  1.0,
                  1.0,
                  1.0,
                  1.0,
                  1.0,
                  1.0,
                  1.0,
                  1.0,
                  1.0,
                  1.0,
                  1.0,
                  1.0
                  }
```

This is only here in case the resource requirement ought to change per month - it is not currently used.   
Need 13 below because SupplyMonth() returns 1-12

const unsigned FemaleTerritoryRange = 8

Minimum territory size from Tine Sussi Hansen data, 2000

Referenced by Vole\_Female::EndStep(), and Vole\_Female::Init().

const unsigned FemaleTerritoryRangeSlope = (MaxFemaleTerrSize-FemaleTerritoryRange)/(MaxWeightF-MinReproWeightF)

only used to create an increasing territory size with age

const int FemNoMature = September

1st september female voles cannot mature after this

Referenced by Vole\_Female::st\_BecomeReproductive().

const double FHabQualThreshold1 = 1.00\*MinFVoleHabQual

Habitat is v.bad below this

const double FHabQualThreshold2 = 1.50\*MinFVoleHabQual

Habitat is OK above this

const double FHabQualThreshold3 = 2.00\*MinFVoleHabQual

Habitat is v.good above this

double g\_extradispmort

Referenced by Vole\_Population\_Manager::Init().

boost::variate\_generator<base\_generator\_type&, boost::uniform\_real<> > g\_rand\_uni

Referenced by Vole\_Male::Dispersal(), Vole\_Female::Dispersal(), Vole\_Base::DoWalking(), Vole\_Base::DoWalkingCorrect(), Vole\_Base::MortalityTest(), Vole\_Male::OnFarmEvent(), Vole\_Female::OnFarmEvent(), Vole\_Female::OnInfanticideAttempt(), Vole\_Female::st\_BecomeReproductive(), and Vole\_Base::Vole\_Base().

double g\_SpeedyDivides[2001]

array of reciprocals to use instead of divding

Referenced by Vole\_Female::BeginStep().

const int GrowStopDate = August

The day it is assumed that grass stops growing

Referenced by Vole\_Male::EndStep(), and Vole\_Female::EndStep().

const double growthperdayF = (MaxWeightF-WeanedWeight)/DaysAtMaxGrowth

g per day during growing for females

Referenced by Vole\_Female::EndStep().

const double growthperdayM = (MaxWeightM-WeanedWeight)/DaysAtMaxGrowth

g per day during growing for males

Referenced by Vole\_Male::EndStep().

const double InfanticideChanceByAge[9] = {0.97,0.86,0.75,0.64,0.54,0.43,0.32,0.21,0.11}

Data from M.arvalis (Heise & Lippke 1997) on infanticide attempts success rate with age of litter

CfgBool l\_pest\_enable\_pesticide\_engine

Referenced by Vole\_Female::BeginStep().

const unsigned MaleMovement[4] = {10,40,70,110}

Max distance between fix distance in Mols radiotracking data was 110m. For the male the distance that he can move increases with size

const int MaleReproductFinish = October

1/10 deduced from Erlinge et al, 1983

const unsigned MaleTerritoryRange = 12

Minimum territory size from Erlinge et al 1990

Referenced by Vole\_Male::DetermineTerritorySize(), and Vole\_Male::Init().

const unsigned MaleTerritoryRangeSlope

**Initial value:**

```
              (MaxMaleTerrSize-MaleTerritoryRange)/(MaxWeightM-MinReproWeightM)
```

only used to create an increasing territory size with age

Referenced by Vole\_Male::DetermineTerritorySize().

const double MaturationChance[5] = {0.020,0.100,0.200,0.500,1.000}

Probability of maturing in each month of age - will do this if 5 months old unless it is winter

Referenced by Vole\_Female::st\_BecomeReproductive().

const unsigned MaxFemaleTerrSize = 16

Maximum territory size for males from Erlinge et al 1990

const unsigned MaxMaleTerrSize = 20

Maximum territory size for males from Agrell et al 1966

const int MaxStarvationDays = 21

The number of days without suitable food the vole will live - this primarily entails risk of predation whilst moving rather than actual startvation.  
The value 21 was fitted based on expert knowledge applied to evaluating individual vole dispersal movements. It has a large influence on colonisation ability!

const int MaxWeightF = 55

The maximum weight of adult females

Referenced by Vole\_Female::EndStep().

const int MaxWeightM = 60

The maximum weight of adult males

Referenced by Vole\_Male::EndStep().

const double MHabQualThreshold1 = 1.00\*MinMVoleHabQual

Habitat is v.bad below this

const double MHabQualThreshold2 = 1.50\*MinMVoleHabQual

Habitat is OK above this

const double MHabQualThreshold3 = 2.00\*MinMVoleHabQual

Habitat is v.good above this

const unsigned MinFemaleMovement = 5

arbitrary minimum movement of 5 - because we need one

const double MinFVoleHabQual = 4.0\*FemaleTerritoryRange\*FemaleTerritoryRange\*1.0

Minimum acceptable habitat quality - assumes that minimum hab qual for survival is 1 \* min female terr size

Referenced by Vole\_Female::Dispersal(), and Vole\_Female::st\_Evaluate\_n\_Explore().

const unsigned MinMaleMovement = 5

arbitrary minimum movement of 5 - because we need one

const double MinMVoleHabQual = 4.0\*MaleTerritoryRange\*MaleTerritoryRange\*1.0

Minimum acceptable habitat quality - assumes that minimum hab qual for survival is 1 \* min male terr size

Referenced by Vole\_Male::CanFeed(), and Vole\_Male::Dispersal().

const int MinReproAgeF = 19

The youngest a female can be reproductive at.

const int MinReproAgeM = 40

The youngest a male can be reproductive at.

Referenced by Vole\_Male::DetermineTerritorySize().

const int MinReproWeightF = 20

The smallest a female can be reproductive at.

Referenced by Vole\_Female::EndStep().

const int MinReproWeightM = 40

The smallest a male can be reproductive at.

Referenced by Vole\_Male::DetermineTerritorySize().

double MoveToLessFavourable

This is specified as a global because it avoids costing time on look-ups.   
This is needed because of the extreme CPU intensive movement functions

Referenced by Vole\_Base::DoWalking(), Vole\_Base::DoWalkingCorrect(), and Vole\_Population\_Manager::Init().

const unsigned TheGestationPeriod = 21

21 days gestation

Referenced by Vole\_Female::st\_Mating().

const int Vector\_x[8] = { 0, 1,1,1,0,-1,-1,-1}

used to define 8 directions - this is the x dimension

Referenced by Vole\_Base::DoWalking(), Vole\_Base::DoWalkingCorrect(), and Vole\_Base::Escape().

const int Vector\_y[8] = { -1,-1,0,1,1, 1, 0,-1}

used to define 8 directions - this is the y dimension

Referenced by Vole\_Base::DoWalking(), Vole\_Base::DoWalkingCorrect(), and Vole\_Base::Escape().

const double VoleHarvestMort = 0.25

Farm operation Mortality

const double VoleHerbicicideMort = 0.0

const double VoleInsecticideMort = 0.0

const unsigned VoleMoveInterval = 30

const double VolePigGrazingMort = 0.25

Farm operation Mortality

const double VoleSoilCultivationMort = 0.75

Farm operation Mortality

const double VoleStriglingMort = 0.50

Farm operation Mortality

const unsigned WeanedAge = 13

Age at weaning

Referenced by Vole\_Female::st\_Lactating().

const int WeanedWeight = 5

Referenced by Vole\_Male::Init(), and Vole\_Female::Init().

---

Generated on Thu Jan 22 14:13:45 2009 for ALMaSS ODDox by 
 1.5.6
